# Aortic dissection diagnosed on stroke computed tomography protocol: a case report

**DOI:** 10.1186/s13256-021-02850-1

**Published:** 2021-05-26

**Authors:** Takami Usui, Kazufumi Suzuki, Hiroshi Niinami, Shuji Sakai

**Affiliations:** 1 Department of Diagnostic Imaging and Nuclear Medicine, Tokyo Women’s Medical University, 8-1, Kawada-cho, Shinjuku-ku, Tokyo, 162-8666 Japan; 2 Department of Cardiovascular Surgery, Tokyo Women’s Medical University, 8-1, Kawada-cho, Shinjuku-ku, Tokyo, 162-8666 Japan

**Keywords:** Computed tomography perfusion, Acute cerebral infarction, Stroke, Acute aortic dissociation

## Abstract

**Background:**

Aortic dissection is one of the causes of stroke. Because cerebral infarction with aortic dissection is a contraindication to intravenous recombinant tissue plasminogen activator (rt-PA) therapy, exclusion of aortic dissection is necessary prior to its administration. However, imaging takes time to provide a diagnosis, possibly causing delays in surgical treatment.

**Case presentation:**

A 65-year-old Japanese female patient was transported to the hospital for a suspected stroke, with back pain and left upper and lower extremity palsy which occurred while eating. Upon arrival at the hospital, the left lower limb paralysis had improved, but the left upper limb paralysis remained. Right back pain had also developed. A plain head computed tomography (CT) scan performed 110 minutes after onset showed no acute bleeding or infarction. Subsequent CT perfusion (CTP) showed acute perfusion disturbance in the right hemisphere without infarction, known as ischemic penumbra. The four-dimensional maximum-intensity projection image reconstructed from CTP showed a delayed enhancement at the right internal carotid and right middle cerebral arteries compared to the contralateral side, suggesting a proximal vascular lesion. Contrast helical CT from the neck to abdomen revealed an acute aortic dissection of Stanford type A with false lumen patency. The dissection extended to the proximal right common carotid artery. The patient underwent an emergency total arch replacement and open stent graft. After recovering well, the patient was ambulatory upon discharge from the hospital. The combination of plain head CT, CTP, and helical CT scan from the neck to abdomen enabled us to evaluate for stroke and aortic dissection within a short amount of time, allowing for early therapeutic intervention.

**Conclusions:**

When acute stroke is suspected due to neurological deficits, plain head CT is the first choice for imaging diagnosis. The addition of cervical CT angiography can reliably exclude stroke due to aortic dissection. CTP can identify ischemic penumbra, which cannot be diagnosed by plain head CT or diffusion-weighted magnetic resonance imaging. These combined stroke CT protocols helped us avoid missing an aortic dissection.

## Background

Aortic dissection is one of the causes of stroke, with a reported frequency of 0.3–10% [1–7]. Performing an imaging diagnosis may take some time, thus delaying treatment. Nevertheless, time must be spent on ruling out aortic dissection before administering intravenous recombinant tissue plasminogen activator (rt-PA) therapy. Cerebral infarction with aortic dissection is a contraindication to intravenous rt-PA therapy, and it has been reported to have a lethal course in such patients [2, 8]. Thus, aortic dissection should be ruled out prior to administration. Rapid and reliable diagnostic methods are essential.

## Case presentation

**Fig. 1:**
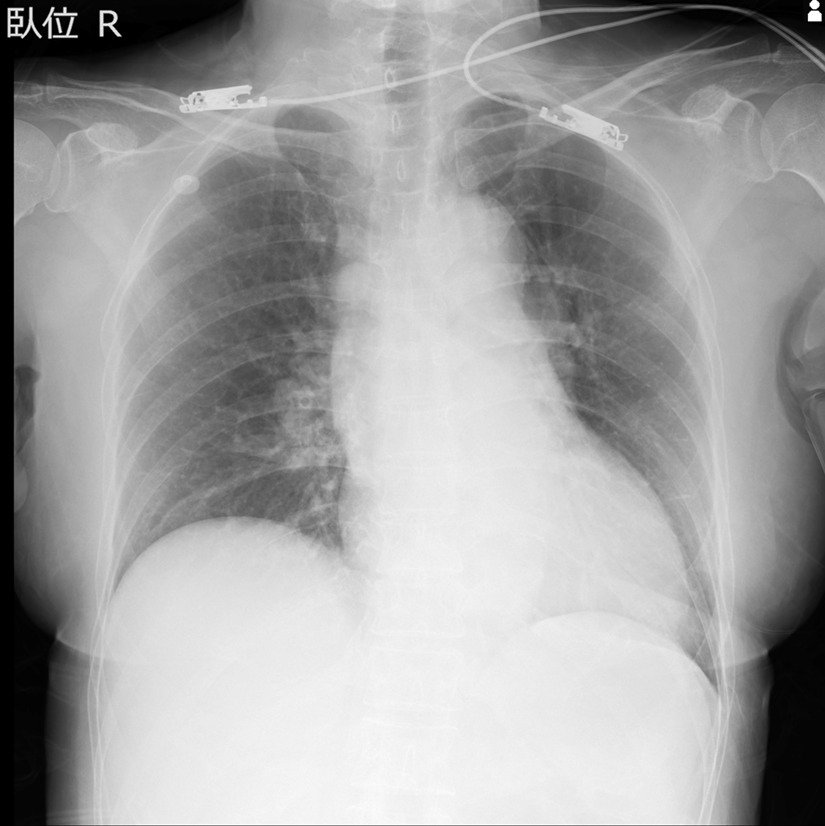
The patient’s chest radiograph shows no enlargement of the mediastinum. There is no calcification of the aortic wall or pleural effusion

**Fig. 2:**
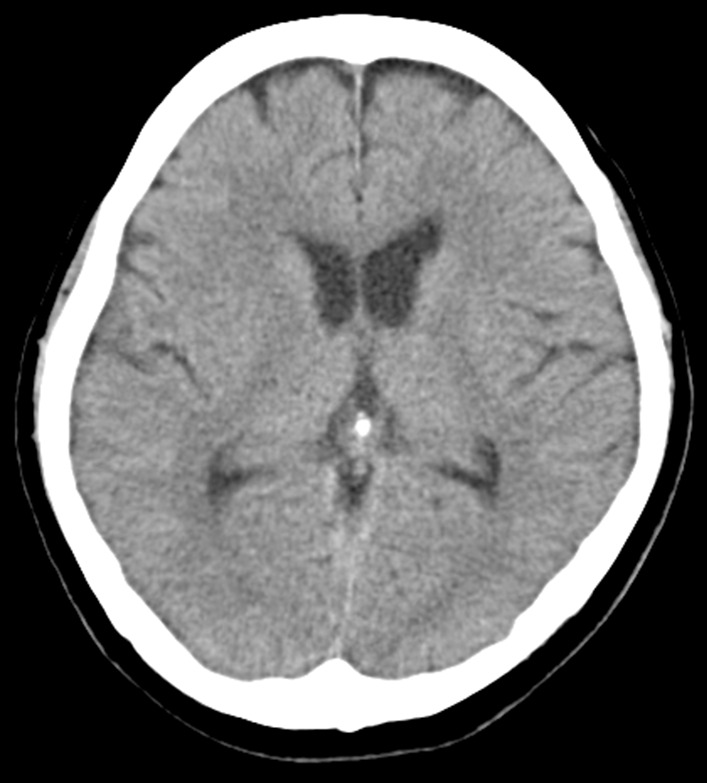
A plain head computed tomography scan does not reveal any sign of acute infarction or bleeding

**Fig. 3:**
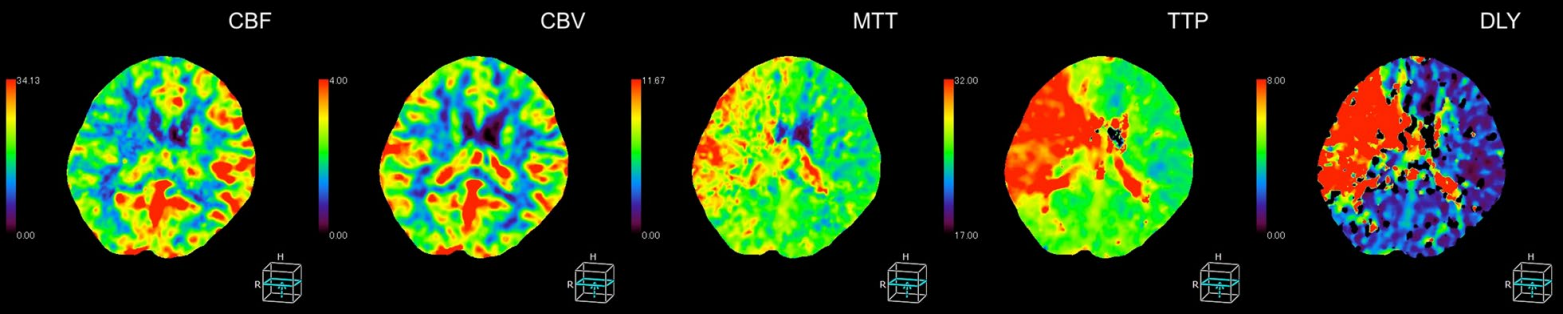
On computed tomography perfusion scan, delay (DLY), time to peak (TTP), and mean transit time (MTT) are elongated. Cerebral blood flow is decreased, but cerebral blood volume (CBV) is preserved in the region of the right middle cerebral artery. The lesions with elongated DLY, TTP, and MTT may indicate major vessel occlusion. An area with decreased CBV is diagnosed as an infarction, which is irreversible ischemic tissue damage. Because CBV remained almost normal, we determined that the patient had a large ischemic penumbra and no infarction yet in this case

**Fig. 4:**
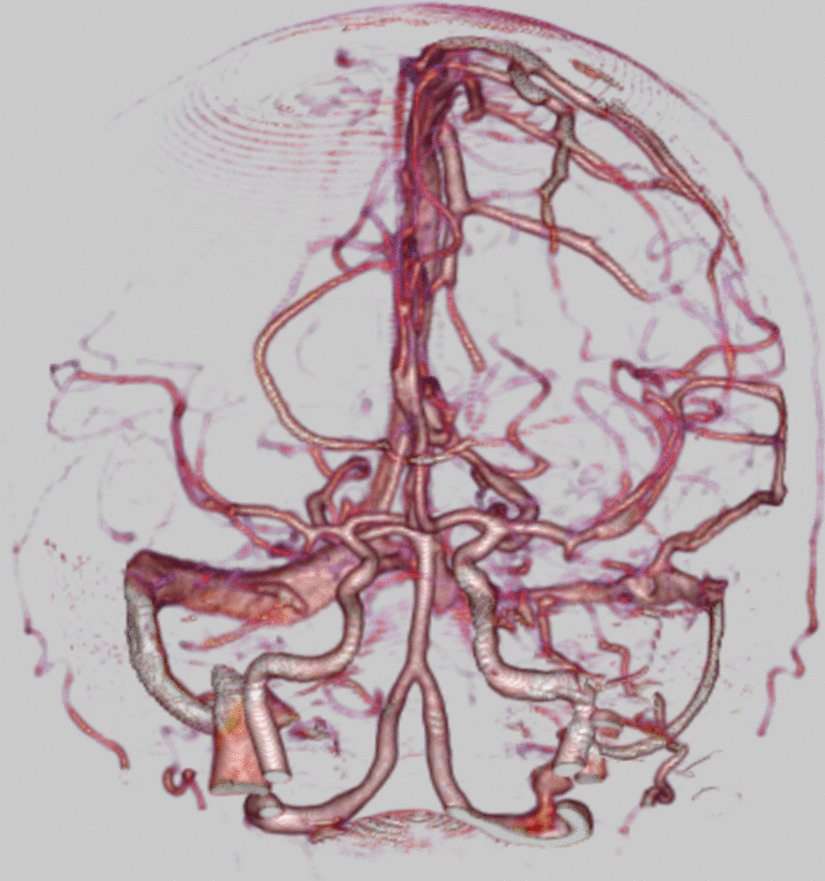
Computed tomography angiography reconstructed from computed tomography perfusion scan shows the right middle cerebral artery poorly depicted. The right internal carotid artery appears to be intact

**Fig. 5:**
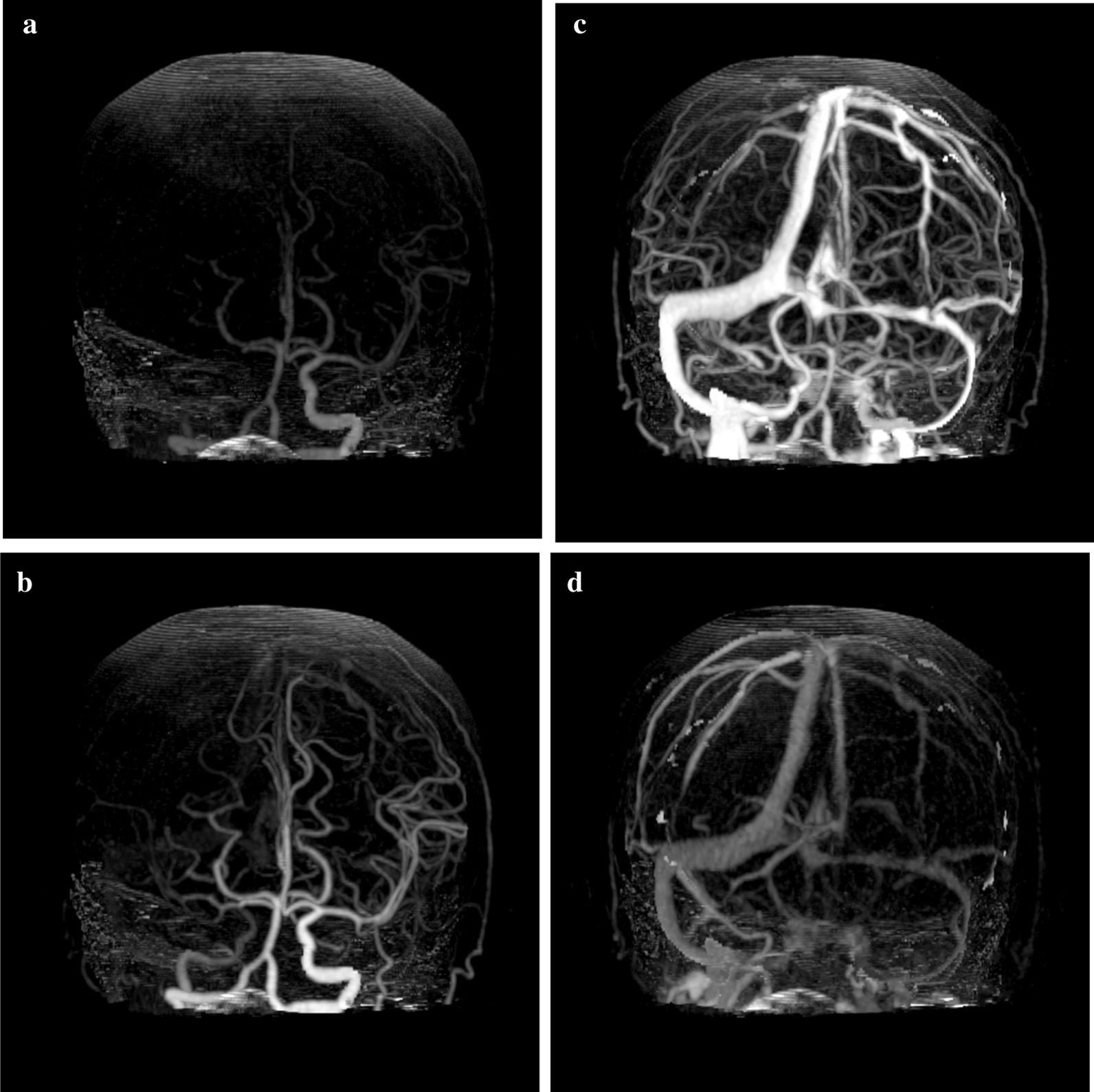
Four-dimensional maximum-intensity projection image reconstructed from computed tomography perfusion scan. **a**,** b** The right internal carotid artery and the right middle cerebral artery indicate delayed enhancement compared to the contralateral side. **c** In the late phase, the distal middle cerebral artery also exhibits delayed enhancement, and no stenosis or obstruction is observed, suggesting a proximal vascular lesion.** d** No venous abnormality was seen in venous phase

**Fig. 6:**
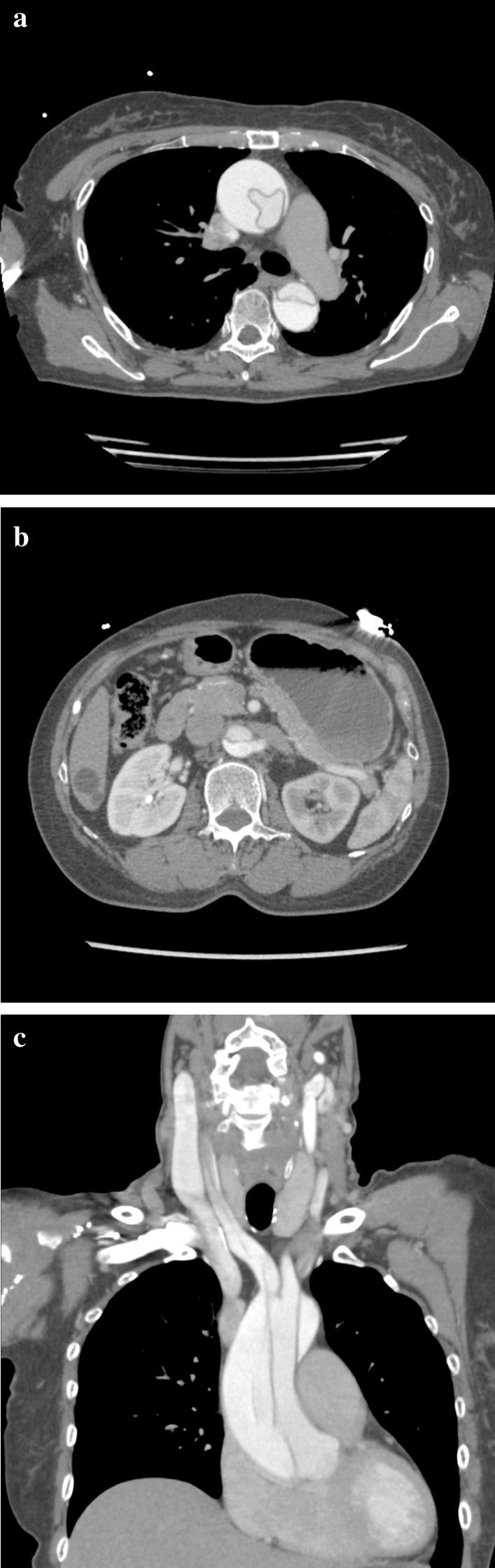
Contrast computed tomography from the neck to abdomen was taken using a contrast medium. **a** A cross section at the level of the thoracic aorta revealed an acute aortic dissection of Stanford type A with false lumen patency. **b** A cross section of the aorta to the level of the renal artery bifurcation. The left renal artery is perfused by the false lumen. **c** Coronal reconstruction section of the neck. The dissection extends to the proximal right common carotid artery, with narrowing of the lumen of the right internal carotid artery

A 65-year-old Japanese female with a history of hyperlipidemia experienced palpitations and uncertain chest discomfort while eating, before developing left upper and lower limb paralysis. During emergency transport, her blood pressure dropped to 62 mmHg, and right joint paralysis developed. She was transported to our hospital 35 minutes after onset with an additional presentation of right back pain. Neurological severity was rated as National Institutes of Health Stroke Scale (NIHSS) 2 (left upper limb paralysis and right conjugate deviation). There was no heart murmur. Left–right difference in blood pressure at the emergency department was less than 20 mmHg (right upper limb 77/54 mmHg, left upper limb 87/45 mmHg, right lower limb 102/92 mmHg, left lower limb 94/51 mmHg). No elevation in white blood cells or C-reactive protein was noted, and there were no findings suggestive of acidosis. d-dimer was mildly elevated at 6.3 μg/mL. Serum creatinine and estimated glomerular filtration rate (eGFR) were 1.11 mg/dL and 38.6 mL/minute/1.73 m^2^, respectively, and renal function was not impaired to the extent that contrast media could not be used. An echocardiogram was not performed. Rehydration was started, and there was no hemodynamic deterioration. A chest radiograph taken 110 minutes after onset showed no enlargement of the aorta or mediastinum (Fig. 1). A plain head CT scan did not suggest acute infarction or bleeding (Fig. 2). However, on CT perfusion (CTP), the right middle cerebral artery region showed an elongation of delay (DLY) and increases in time to peak (TTP) and mean transit time (MTT). Cerebral blood flow (CBF) was decreased, but cerebral blood volume (CBV) was preserved (Fig. 3). Due to the absence of early CT signs in the plain head CT, we determined that the patient had ischemic penumbra. On the CT angiography (CTA) reconstructed from the arterial phase of the CTP scan, the right middle cerebral artery was poorly depicted (Fig. 4). However, the four-dimensional maximum-intensity projection (4D MIP) image reconstructed from CTP showed a delayed enhancement of both the right internal carotid artery and the right middle cerebral artery. In the late phase, the distal portion of the right middle cerebral artery was well depicted, and no stenosis or obstruction was observed at the intracranial artery, suggesting a proximal vascular lesion (Fig. 5). A contrasted helical CT scan from the neck to abdomen was performed using contrast medium residue soon after CTP imaging. This revealed an acute aortic dissection of Stanford type A with false lumen patency, from the base of the aorta to the level of the renal artery bifurcation. The dissection extended to the proximal right common carotid artery, and the true lumen of the right internal carotid artery was narrowed. The left renal artery was supplied by the false lumen (Fig. 6). The brain tissue status on CTP was reversible ischemia, not irreversible infarction, and the patient was deemed to be eligible for emergency surgery, which included total arch replacement and open stent graft insertion approximately 3.5 hours after the onset of illness. The left upper limb paralysis had recovered to a manual muscle test (MMT) grade of about 4–5 after the surgery. The patient was ambulatory upon discharge from the hospital 23 days after the onset.

## Discussion and conclusions

Intravenous rt-PA therapy is contraindicated in cases of cerebral infarction with aortic dissection. When rt-PA is considered in acute ischemic stroke, aortic dissection should be excluded. Amr *et al*. noted that brain hypoperfusion alone should not be a contraindication for urgent surgical treatment, regardless of initial clinical neurological severity [8]. Ultrasonography such as point-of-care ultrasound is usually performed to arrive at an early diagnosis [9], but unfortunately it was not performed in this case. Although ultrasonography can be used for an early diagnosis, it is first necessary to understand the exact type and condition of aortic dissection before surgery in order to determine whether emergency surgery should be performed. In this case, we were misled by the importance of neurological findings despite the presence of symptoms suggestive of acute aortic dissection. However, by using the stroke CT protocol, we arrived at the correct diagnosis of acute aortic dissection with a single examination modality. We believe that contrast CT is useful in all cases when stroke is suspected.

When acute stroke is suspected due to neurological deficits, plain head CT is the first choice of imaging for diagnosis [10]. CTP can evaluate ischemic penumbra, which cannot be diagnosed using plain head CT or diffusion-weighted magnetic resource imaging (DW-MRI). Cerebral circulatory disturbance in acute aortic dissection may cause impaired consciousness and paralysis, and surgery is indicated when the disease complications can be alleviated or its progression can be controlled by surgery [11, 12]. In this case, CTP revealed that the condition of the brain tissue was reversible ischemia, not irreversible infarction.

Herein, we present our stroke CT protocols as they are currently employed. First, plain head CT is used to determine the presence of cerebral hemorrhage and evaluate early CT signs. Next, a CTP is taken using 50 mL of contrast agent to check for ischemic penumbra. The addition of cervical CTA using 40 mL of contrast agent can reliably exclude stroke due to aortic dissection. An aorta study may be taken instead with helical CT from the neck to the abdomen or mid-thigh level to prepare for catheterization. This usually takes only 10–15 minutes, and in this way, maximum imaging can be achieved in a short amount of time. In this case, the time from scouting to contrast CT imaging was 24 minutes. The delay may have been because the case came in at night and was received by a technician unfamiliar with our stroke CT protocols. Nevertheless, although the examination took longer than usual, the patient was still diagnosed within 90 minutes after the onset of symptoms and was able to undergo emergency surgery in about 3.5 hours.

This case may seem like a normal course of action to some experts, but it highlights some important issues. Stroke guidelines recommend the use of CTP between 6 and 24 hours after onset to determine indications for revascularization in ischemic stroke [10]. However, aortic dissection complications and ischemic penumbra can be assessed simultaneously using contrast CT as indicated by the appropriate stroke protocols, as in the present case. This approach can be of benefit in patients up to 6 hours from the onset of stroke.

However, some problems still need to be addressed before CTP can become the standard. There is a risk of developing an allergy from the use of iodine contrast agents in some patients. In addition, an optimizing protocol is required, such as the use of iterative reconstruction methods and the optimization of intermittent scans to reduce radiation exposure with a multiphase scan protocol.

Aortic dissection is associated with a high mortality rate and poor prognosis, but if it is diagnosed and treated quickly, a positive turnaround may be possible [8, 13]. The stroke CT protocol may have advantages in diagnosing stroke and aortic dissection due to its short examination time [14–17].

## Data Availability

The datasets supporting the conclusions of this article are included within the article.

## Abbreviations

CBF: Cerebral blood flow
CBV: Cerebral blood volume
CTA: Computed tomography angiography
CTP: Computed tomography perfusion
DLY: Delay
NIHSS: National Institutes of Health Stroke Scale
MMT: Manual muscle test
MTT: Mean transit time
TTP: Time to peak
4D MIP: Four-dimensional maximum-intensity projection
